# A Deep-Learning-Based Detection Approach for the Identification of Insect Species of Economic Importance

**DOI:** 10.3390/insects14020148

**Published:** 2023-01-31

**Authors:** Michael Tannous, Cesare Stefanini, Donato Romano

**Affiliations:** 1 The BioRobotics Institute, Sant’Anna School of Advanced Studies, Viale Rinaldo Piaggio 34, 56025 Pontedera, Italy; 2 Department of Excellence in Robotics and AI, Sant’Anna School of Advanced Studies, 56127 Pisa, Italy

**Keywords:** deep learning, AI, agtech, integrate pest management, machine learning, tephritid, olive fruit fly, Mediterranean fruit fly, monitoring, real-time classification

## Abstract

**Simple Summary:**

This study aims at developing a machine-learning-based classification approach to recognize insect species of economic importance. Two tephritid pest species with similar shape and locomotory patterns (e.g., the Mediterranean fruit fly *Ceratitis capitata*, and the olive fruit fly *Bactrocera oleae*) were used as model organisms. The proposed method, based on a convolutional neural network (CNN), accurately detects and discriminates moving *C. capitata* and *B. oleae* adult individuals in real-time. These results importantly contribute to the development of autonomous pest monitoring methods, to intervene with tailored measures instantaneously and remotely. Overall, this study promotes sustainable and efficient crop protection approaches based on integrated pest management and precision techniques.

**Abstract:**

Artificial Intelligence (AI) and automation are fostering more sustainable and effective solutions for a wide spectrum of agricultural problems. Pest management is a major challenge for crop production that can benefit from machine learning techniques to detect and monitor specific pests and diseases. Traditional monitoring is labor intensive, time demanding, and expensive, while machine learning paradigms may support cost-effective crop protection decisions. However, previous studies mainly relied on morphological images of stationary or immobilized animals. Other features related to living animals behaving in the environment (e.g., walking trajectories, different postures, etc.) have been overlooked so far. In this study, we developed a detection method based on convolutional neural network (CNN) that can accurately classify in real-time two tephritid species (*Ceratitis capitata* and *Bactrocera oleae*) free to move and change their posture. Results showed a successful automatic detection (i.e., precision rate about 93%) in real-time of *C. capitata* and *B. oleae* adults using a camera sensor at a fixed height. In addition, the similar shape and movement patterns of the two insects did not interfere with the network precision. The proposed method can be extended to other pest species, needing minimal data pre-processing and similar architecture.

## 1. Introduction

The recent rapid progress in Artificial Intelligence (AI) and automation is producing a new wave of technological advancement with tangible impact on social, health, industrial, and environmental contexts [1,2,3]. AI provides robust applicability to complex problems, with performances, in some scenarios, challenging human results. Agriculture represents an application field of crucial importance for AI, due to the benefits that machine learning strategies may provide to face challenges related to pest and disease monitoring, weed management, chemicals use, irrigation issues, yield prediction, precision livestock farming, and more [2,4,5,6]. AI techniques provide the best fitting solution for specific agricultural problems that are generally highly dynamic and cannot be generalized to propose a common solution [2,7]. 

Crop protection is one of the most expensive practices in the agribusiness, often related to improper strategies adopted, as well as to the impossibility of adequately recognizing and preventing severe parasite infestations and pathogen infections [8,9]. In addition, an increasing environmental concern and public demand for the reduction of toxic insecticides use, make pest management even more challenging [10]. Integrated pest management (IPM) aims at ensuring more sustainable and efficient crop protection programs based on adequate strategies to control pests [8]. One of the IPM’s core components is represented by monitoring pests’ activity and density [11]. However, monitoring operations are still mainly based on human experts that analyse traps *in loco*, or their digital images, to recognize and count pests [12]. This monitoring approach is labour intensive, time demanding, and expensive [13]. Furthermore, the unavailability of a standardized counting process frequently makes monitoring operations error prone [14].

The option of autonomously and remotely monitoring pest organisms is gaining a momentum due to the advantage to intervene in protectingcrops in real time [15,16,17,18]. In several previous studies, insect models have been used, since specimens are generally well preserved, and their images can be captured at high resolution in ideal laboratory conditions [19,20,21,22]. In other research, insects collected in nature have been classified in laboratory conditions [23,24,25]. Image quality was worse than those of the specimen’s case, but thanks to the laboratory environment, it was possible to adjust several experimental settings. Different machine learning models have been used, such as support vector machines (SVM) [21], artificial neural networks (ANN) [21,26], k-nearest neighbours (KNN) [27], and ensemble methods (e.g., adaptive boosting [28,29]). In a general perspective, the state of the art for insect ecology investigation and monitoring includes four main technologies [30]: computer vision, acoustic monitoring [31], radar technologies [32,33], and molecular methods [34].

However, beside the morphology of stationary or immobilized animals, other features related to living animals behaving in the environment (e.g., walking trajectories, different postures, etc.) have been overlooked so far. Developing a technology considering these features can strongly contribute to improve automatic monitoring in real scenarios. Furthermore, localizing and classifying insects that naturally behave in the environment, can pave the way to the development of a new generation of monitoring stations that are more selective than traditional sticky traps. For instance, Bjerge et al. [35] constructed a system using a deep learning software that performs real-time classification and tracking of pollinators. Unfortunately, sticky traps also capture non-target and/or beneficial organisms [36,37,38,39], with obvious negative effects on biodiversity and ecosystem stability.

Herein, we developed a detection method based on a convolutional neural network (CNN) that can accurately classify living true fruit flys’ species (Diptera: Tephritidae) moving and constantly changing their posture in real-time. Particularly, an artificial neural network (ANN) was trained on two different species belonging to the family Tephritidae: the Mediterranean fruit fly, *Ceratitis capitata* Wiedemann, a polyphagous pest attacking more than 200 fruit species [40]; and the olive fruit fly *Bactrocera oleae* (Rossi), that is the major pest of commercial olives worldwide [41]. Our approach can be extended to other pest species, with minimal data pre-processing. In a broader context, the technology can be used for a wide range of applications with other species of economical and medical importance, such as pollinators and mosquitoes [42].

## 2. Materials and Methods

### 2.1. Ethics Statement

This research is compliant with the guidelines for the treatment of animals in behavioral research and teaching [43], as well as with the Italian (D.M. 116192) and the European Union regulations [44].

### 2.2. Animal Rearing

*C. capitata* and *B. oleae* adult flies were maintained in separate cylindrical PVC cages under controlled conditions (21 ± 1 °C, 55 ± 5% relative humidity, 16:8 h light:dark) at the BioRobotics Institute. Adults were fed on a dry diet of yeast extract and sucrose mixture, at a ratio of 1:10 (*w*:*w*), while a cotton wick provided water.

### 2.3. Images Acquisition Setup

Flies were individually transferred in a transparent petri dish (60 mm diameter) turned upside down avoiding insects’ escape. An equal proportion of males and females for each species were tested. Although sexual dimorphism exists in both species, the colours and morphology between the two species are clearly distinguishable regardless of their sexes.

**Figure 1:**
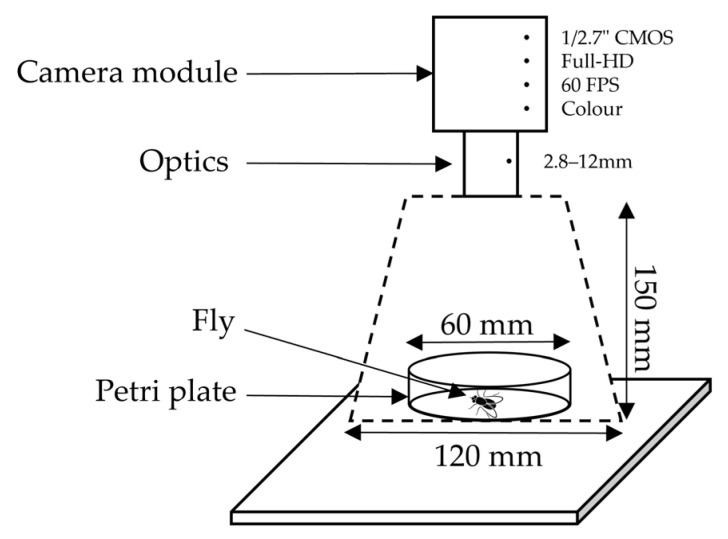
Experimental setup for images acquisition with the camera pointing to the fly inside a petri dish.

The petri dish was framed by an image sensor (specifically, camera module with 1/2.7” CMOS sensor, Full-HD maximum resolution, 60 FPS, and coloured), mounting fixed 2.8–12 mm optics (i.e., without automatic focus), Figure 1. Flies were allowed to move freely inside the plate enabling the camera to capture each insect in different poses and positions in order to build a relevant dataset. In the configuration with maximum resolution (i.e., 1920×1080) 1 pixel covers 0.6 mm.

**Figure 2:**
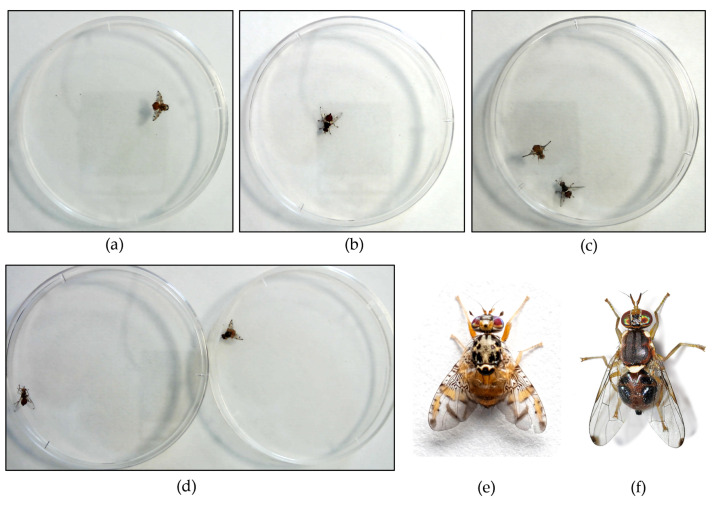
Dataset composition: (**a**) *C. capitata* adult fly in a petri dish; (**b**) *B. oleae* adult fly in a petri dish; (**c**) *C. capitata* and *B. oleae* in the same petri dish (performance set); (**d**) *C. capitata* and *B. oleae* in different petri dishes (performance set). Photographs of (**e**) *C. capitata* adult fly and (**f**) *B. oleae* adult fly.

Flies were recorded both individually and in pairs (i.e., one per each fly) in the same petri plate, without additional illumination other than laboratory lights, Figure 2. Recording time for each video varies from a few seconds up to 3 min depending on the insects’ behaviour. For instance, some recordings were stopped in case the insect remained motionless for several seconds. The use of video recording simplifies the data collection phase, avoiding the set of a timer for image capturing which can lead to blurred images or missing insect’s motion. In addition, video recording is easier to analyse than single images and faster in trimming parts where insects are motionless. Images selection criteria maximizes insects’ different poses and minimizes multiples of similar ones to improve the training process.

Although, *C. capitata* and *B. oleae* adult flies are chromatically and morphologically distinct as shown in Figure 2, differences are reduced in the captured images given the insects’ small size (i.e., 4–6 mm long). Hence, images display two similar insects making the recognition process challenging.

### 2.4. Dataset Creation and Pre-Processing

Frames were extracted from the captured videos by manually labelling the region of interest (ROI) of each fly in multiple poses for high quality annotation. Then, datasets were pre-processed to generate different resolution sizes for a better network generalization. Multi-scale feature extraction is a method to improve the training accuracy of neural network models. This method first scales the input image to several different scales, then performs feature extraction of the image at each scale and finally constructs a feature pyramid using all the extracted scale features and inputs them into the neural network model. In this work, three resolutions were used: 1920 × 1080, 640 × 360 and 416 × 234.

**Figure 3:**
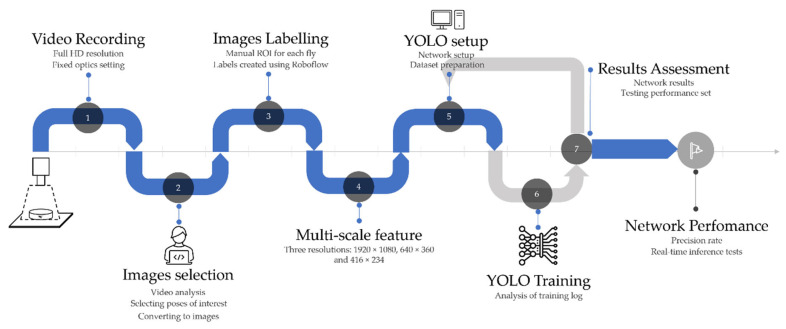
A schematic representation of the developed steps from data capturing (1) and data preprocessing (2–4) towards network setup, training, and performance evaluation (5–7).

Datasets were composed of 912 images split in 70% training set, 20% validation set and 10% testing set. The training set was almost equally distributed with 49% images for *C. capitata* and 51% images for *B. oleae*. Furthermore, frames used for training included only individual insects in the petri dish, the dataset of multiple insects in the same image, called the performance set (i.e., 914 images), was used afterwards to assess the network performance, Table 1. Once results are evaluated using the performance set, validation and testing sets’ distributions are irrelevant. A schematic workflow of the developed approach is shown in Figure 3. 

### 2.5. YOLO Network

Convolutional neural networks of the YOLO (You only look once) family [45,46,47] are one-stage object detection systems computationally inexpensive with good performance metrics and outperforming other target recognition algorithms [48]. These networks were used in a variety of recognition tasks: apple detection during different growth stages [49], uneaten feed pellets detection in underwater images for aquaculture [50], simultaneous detection and classification of breast masses in digital mammograms [51], automatic license plate recognition [52] or even medical face mask detection in COVID-19 scenarios [53]. Compared with the Faster R-CNN network, the YOLO network transforms the detection problem into a regression one without requiring a proposal region. Indeed, the network, using a single CNN for the entire image, divides the input image into sub-regions predicting multiple bounding box coordinates and probabilities of each class directly through regression increasing the detection speed.

YOLOv5 network [54] is a version of the YOLO architecture series. This network model has proven to significantly improve the detection accuracy and the inference speed (the fastest detection speed being up of 140 frames per second); these attributes are of great importance when moving forward in system implementation to bigger datasets and real-time detection. Nevertheless, the size of the weight file related to the target detection network model is small, nearly 90% smaller than the previous YOLOv4, meaning that the YOLOv5 model is lightweight and suitable for deployment to embedded devices implementing real-time detection. For the sake of completeness, YOLOv5 contains four architectures: YOLOv5s, YOLOv5m, YOLOv5l, and YOLOv5x. Each architecture has a different amount of feature extraction modules and convolution kernel. Simplifying, size and parameters number of the model in the four architectures increase in turn. Since, we have two targets to be identified in this study, and the recognition model has high requirements for real-time performance and lightweight properties, we based our experimental architecture on YOLOv5s. The network model size is about 14 MB and its inference time with PyTorch is 2.2 ms.

**Figure 4:**
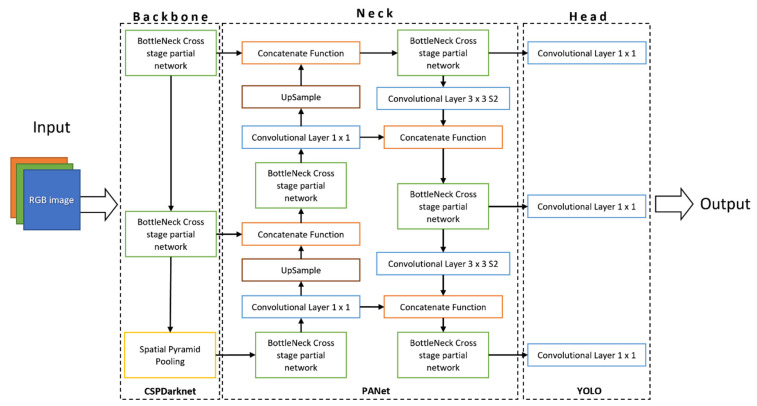
YOLOv5 network architecture split into three main parts: Backbone, Neck, and Head.

The network architecture of YOLOv5, shown in Figure 4, is divided into three main parts: Backbone, Neck and Head, built on CSPDarknet (cross stage partial network into Darknet), PANet (path aggregation network), and YOLO layer, respectively. Images are the input of CSPDarknet for features extraction, then PANet is fed for feature fusion and, lastly, the YOLO layer returns detection results such as class, probability, location, and size.

### 2.6. Network Training and Testing Results

The network is built up by PyTorch [55] and trained on Intel Core i7-7820X, with a 3.60 GHz processor, 32 GB RAM, 500 GB SSD, and 8 GB NVIDIA GTX 1070 using Windows OS. Results are classified as: True Positive (TP), True Negative (TN), False Positive (FP), and False Negative (FN) [56]. TP and TN indicate the truly recognized olive/Mediterranean fruit fly points by each algorithm. Detection errors, instead, are shown through the FP and FN classes which indicate any olive (or Mediterranean) fruit fly identification (or miss), respectively. The binary classification parameters are collected in the so-called confusion matrix that allows the quick visualization of the algorithm performance, shown in Table 2.

Three indices are taken into account to assess each algorithm performance. Firstly, the precision percentage or positive predictive value (PPV) calculated as:

(1) $${\mathrm{PPV}}_{\%}=\frac{\mathrm{TP}}{\mathrm{TP}+\mathrm{FP}}\times 100$$

that gets the highest values when high precision is achieved, meaning that the network returned more relevant than irrelevant results. Another important index is the sensitivity, true positive rate (TPR), or recall defined as

(2) $${\mathrm{TPR}}_{\%}=\frac{\mathrm{TP}}{\mathrm{TP}+\mathrm{FN}}\times 100=100-{\;\mathrm{FNR}}_{\%}$$

where $\mathrm{FN}R_{\%}$ is the false negative rate. 

Finally, the accuracy in unbalanced classes is measured by the F1 score (also F-score or F-measure) defined as:

(3) $$F_{1}=2\cdot \frac{\mathrm{precision}\;\times \;\mathrm{sensitivity}}{\mathrm{precision}\;+\;\mathrm{sensitivity}}=\frac{2\mathrm{TP}}{2\mathrm{TP}\;+\;\mathrm{FP}\;+\;\mathrm{FN}}\;$$

YOLOv5 model evaluation includes the mean average precision (mAP), detection time, intersection over union (IoU), and floating-point operations (FLOPS). IoU calculates the overlap ratio between the boundary box of the prediction (pred), ground-truth (gt) [57]:

(4) $$\mathrm{IoU}=\frac{{\mathrm{Area}}_{\mathrm{pred}}\cap {\mathrm{Area}}_{\mathrm{gt}}\;}{{\mathrm{Area}}_{\mathrm{pred}}\cup {\mathrm{Area}}_{\mathrm{gt}}\;}\;$$

YOLO training options are summarized in Table 3. Uncited network parameters are set to zero. 

## 3. Results

**Figure 5:**
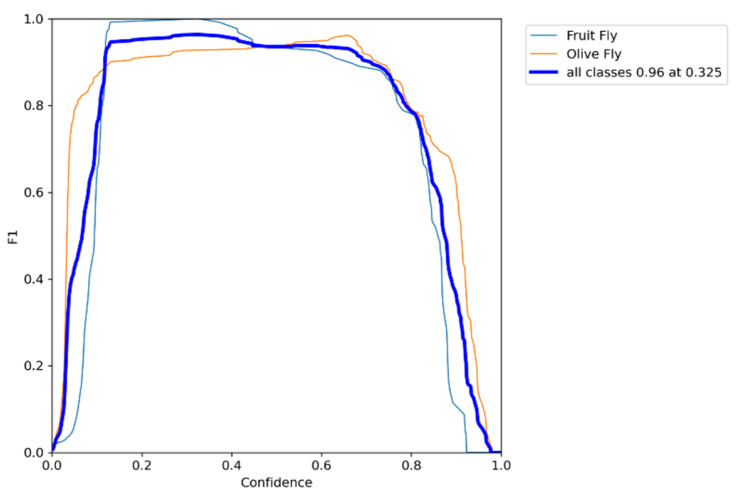
YOLO training F1 score results.

**Figure 6:**
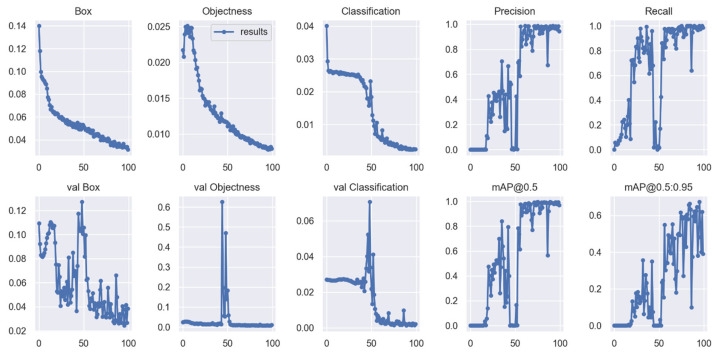
Visualization of various metrics (e.g., precision, recall, mAP_0.5_, etc.) with the number of epochs (i.e., *x*-axis) during training and validation.

**Figure 7:**
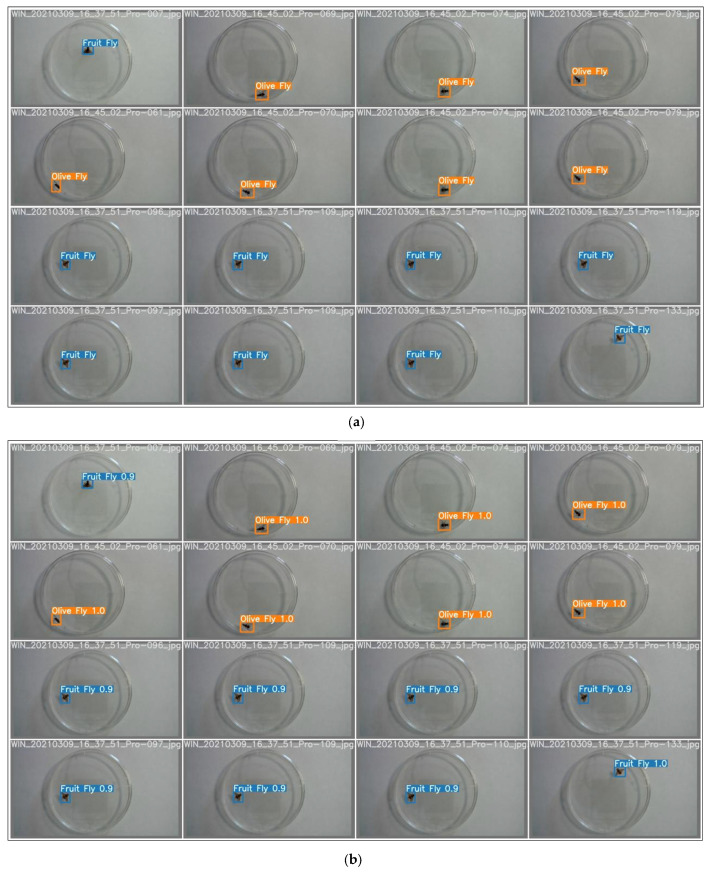
Training results: (**a**) labelled images of the olive fruit fly (i.e., *B. oleae* in the orange box) and the Mediterranean fruit fly (i.e., *C. capitata* in the blue box), (**b**) prediction results of the testing set with inference precision percentage.

**Figure 8:**
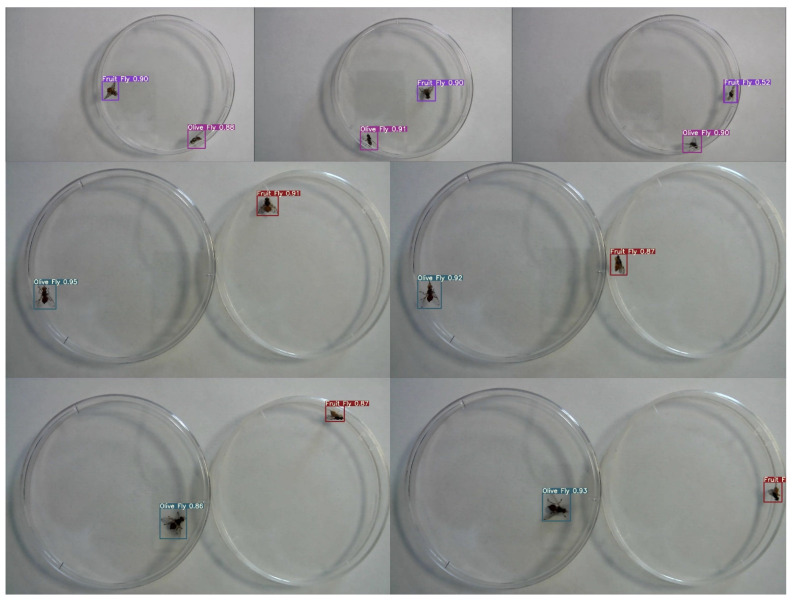
YOLO testing results using performance set. Colored boxed indicated the olive fruit fly (i.e., pink and green) and the Mediterranean fruit fly (i.e., purple and red) recognized from the network along with the precision rate percentage.

The training confusion matrix and F1 score are shown in Table 4 and Figure 5, respectively. In some of these figures, species names are abbreviated for convenience only with fruit fly (i.e., Mediterranean fruit fly) and olive fly (i.e., olive fruit fly). Training results, however, are summarized in Figure 6. In particular, the progressions of various metrics are shown such as box, objectness and classification during training and validation; metrics such as precision, recall, and mAP_0.5_ after each epoch are also plotted. The network performed very well in terms of precision (95%), recall (97%), and mAP_0.5_ (95%). Examples of the testing set results are presented in Figure 7, showing the network precision in classifying labelled images. The YOLO network was trained with a dataset composed of the olive fruit fly and the Mediterranean fruit fly separately (i.e., training set in Table 1). Afterwards, the network was tested with the performance set (i.e., dataset with two flies in the same image, Table 1). Figure 8 shows a visualization of some output from the network on tested images of the performance set. The precision rate of the performance set was about 93% in total for both datasets including two insects in the same petri dish or in separated ones.

## 4. Discussion

**Figure 9:**
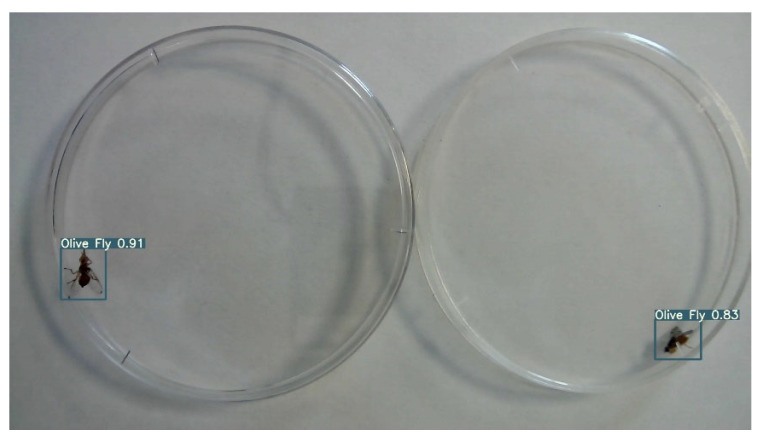
Detection failed to recognize the Mediterranean fruit fly (right) in an angular pose (slightly blurred) of the insect’s body.

Results showed the feasibility of using the proposed method for real-time and accurate detection of *C. capitata* and *B. oleae* adult flies. In the experimental setup, the network performed good, as shown in Figure 6, with precision (95%), recall (97%), and mAP_0.5_ (95%). The software based on YOLOv5s architecture requires 14 MB of memory with PyTorch inference time of 2.2 ms. Hence, a compatible on-field hardware could be a DSP or a microcontroller with single core processor, and a relatively small memory capacity. The comparison between these two fly species, belonging to the same family, at a fixed camera height demonstrated that even insects with similar visual and motor patterns could be detected from YOLO with a good precision rate (about 93%). Nevertheless, the similarity challenges the network in some angular poses of the insect. For instance, in Figure 9 the network was unable to recognize the Mediterranean fruit fly, classifying it as an olive fruit fly with high precision. This image is not easy to classify, even manually by an operator, and only insect’s sizing may help us in the classification. Insects with a small size and morphologically similar (on a macro scale) are still challenging for deep learning techniques.

The white background enhances image contrast in both training and inference phases for these species with body colours prevalently black and grey. In general, the background colour is an important parameter to tune both for enhancing and for filtering some insects, making them invisible for the network inference. For instance, if the monitoring scope is focused on insects with dominant white colour, then white background will probably be a big issue for the network training. 

The results obtained with YOLO suggest that innovative systems for monitoring pests’ population dynamic can be developed relying on machine learning techniques. These systems may include a network of low-cost microcontrollers connected to a CMOS sensor presenting a configuration similar to that in Figure 1. In addition, the integration of a wireless communication module can enable information exchange with field monitoring platforms. Energy consumption has to be estimated and it is strongly related to the selected hardware. HHowever, micro-solar panels (electrical power from 0.3 W to 3 W roughly) can provide a valid solution at least during warmer seasons.

This study may promote the development of innovative approaches based on a distributed network of automatic monitoring platforms in the field to investigate the population dynamic of key insect species. This would contribute to increase sustainability and efficiency of control measures, and reduce negative effects on non-targeted or even beneficial organisms. This approach can be extended to other species of interest, with minimal data pre-processing and similar architecture.

## 5. Conclusions

A CNN based on the YOLO network was used as a detection method to classify in real-time living fruit fly species (Diptera: Tephritidae) while moving and constantly changing their posture. We trained an artificial neural network on two different species belonging to the family Tephritidae: the Mediterranean fruit fly *C. capitata*, a highly polyphagous phytophagous widely distributed throughout the world; and the olive fruit fly *B. oleae*, that is the major pest of commercial olives worldwide.

The comparison between these two fly species using a fixed camera height demonstrated that even insects with similar shape and movement patterns could be detected by the network with a precision rate of about 93%. The software based on YOLOv5s architecture requires 14 MB of memory with PyTorch inference time of 2.2 ms. Low-cost monitoring systems can be developed with minimal hardware, creating a sensing network to monitor the insects’ population dynamic in the field.

Our approach can be extended to other pests, pollinators, and hematophagous species with minimal data pre-processing. Further studies will focus on tests development in field conditions with a custom hardware configuration.

## Figures and Tables

**Table 1:** Dataset composition, size, and type distribution.

Dataset Composition	Dataset Size	Dataset Type
*C. capitata*	309	Training set
*B. oleae*	330	Training set
*C. capitata*	66	Validation set
*B. oleae*	117	Validation set
*C. capitata*	63	Testing set
*B. oleae*	27	Testing set
*C. capitata* and *B. oleae*	914	Performance set

**Table 2:** Confusion matrix table layout.

		Predicted Class	
		Positive	Negative
**Actual class**	Positive	TP	FN
	Negative	FP	TN

**Table 3:** YOLO training options and parameters setting.

Parameter	Value	Parameter	Value
lr0	0.01	lrf	0.2
momentum	0.973	weight decay	0.0005
warmup epochs	3.0	warmup momentum	0.8
warmup bias lr	0.1	box	0.05
cls	0.5	cls_pw_	1.0
obj	1.0	obj_pw_	1.0
IoU_t_	0.2	anchor_t_	4.0
hsv_h_	0.015	hsv_s_	0.7
hsv_v_	0.4	translate	0.1
scale	0.5	fliplr	0.5

**Table 4:** YOLO training confusion matrix results.

		True Condition	
	Training	Mediterranean Fruit Fly	Olive Fruit Fly
Predicted Condition	Mediterranean Fruit Fly	TP=213 $\left(100\%\right)$	FP=16 $\left(0\%\right)$
	Olive Fruit Fly	FN=11 $\left(93\%\right)$	TN=698 $\left(5\%\right)$

## Data Availability

Data are available on request.
